# Social support and hospitalization in the elderly: investigating the role of frailty trajectories

**DOI:** 10.1093/eurpub/ckag011

**Published:** 2026-02-16

**Authors:** Paola Scarcella, Fausto Ciccacci, Annamaria Doro Altan, Olga Madaro, Leonardo Emberti Gialloreti, Stefano Orlando, Clara Donnoli, Michele Bisogno, Fabio Riccardi, Rita Cutini, Giuseppe Liotta

**Affiliations:** Human Sciences Department, LUMSA University, Rome, Italy; Biomedicine and Prevention Department, University of Rome Tor Vergata, Rome, Italy; Link Campus University, Rome, Italy; Community of Sant’Egidio—Long Live the Elderly! Program, Rome, Italy; Biomedicine and Prevention Department, University of Rome Tor Vergata, Rome, Italy; Biomedicine and Prevention Department, University of Rome Tor Vergata, Rome, Italy; Biomedicine and Prevention Department, University of Rome Tor Vergata, Rome, Italy; Biomedicine and Prevention Department, University of Rome Tor Vergata, Rome, Italy; Biomedicine and Prevention Department, University of Rome Tor Vergata, Rome, Italy; GEPLI Department, LUMSA University, Rome, Italy; Biomedicine and Prevention Department, University of Rome Tor Vergata, Rome, Italy

## Abstract

Biopsychosocial frailty, integrating physical, psychological, and social dimensions, significantly affects health outcomes in older adults. Hospitalization, a major contributor to healthcare burden, is strongly associated with frailty. However, the role of socioeconomic determinants within frailty trajectories remains insufficiently explored. This study aimed to evaluate the association between biopsychosocial frailty trajectories and hospitalization rates, with a focus on social determinants. We conducted a retrospective cohort study involving 6086 individuals (mean age 83.6 ± 4.9 years; 65.9% women). They underwent serial frailty assessments between 2016 and 2024 using the Short Functional Geriatric Evaluation (SFGE). Frailty trajectories were categorized as improved, stable, or worsened. Hospitalization rates were analyzed through parametric/non-parametric tests and negative binomial regression models adjusted for age, baseline frailty, and psycho-physical status. Hospitalization rates increased with frailty severity: 84‰ in robust, 97‰ in pre-frail, 149‰ in frail, and 136‰ in very frail individuals (*P* < 0.001). Improved or stable financial conditions significantly reduced hospitalization risk (rate ratio [RR] 0.24 and 0.41, respectively), as did stable or restored informal support networks (RR 0.45 and 0.79, respectively). Improved living arrangements were also associated with reduced hospital admissions. Robust and pre-frail individuals accounted for ∼50% of all admissions. Social and economic stability are key protective factors against hospitalization in older adults, independent of physical frailty. Community-based interventions addressing social isolation and financial vulnerability could substantially reduce hospital admissions, particularly among robust and pre-frail individuals. A holistic approach integrating social, economic, and physical frailty dimensions is recommended to optimize public health strategies for aging populations.

## Introduction

Frailty is a clinically recognizable state of increased vulnerability resulting from age-associated decline across multiple physiological systems, reducing the capacity to cope with acute stressors [1, 2]. Two conceptual frameworks have shaped contemporary frailty research: the frailty phenotype, which identifies frailty through physical markers such as weakness, slowness, and unintentional weight loss [3], and the deficit accumulation approach, which conceptualizes frailty as the cumulative effect of health deficits across multiple domains [4].

Beyond these predominantly physical conceptualizations, emerging research recognizes frailty as a biopsychosocial phenomenon. Biopsychosocial frailty represents a complex and multidimensional condition involving biological, psychological, and social aspects, significantly affecting the quality of life and adaptive capacity of individuals [5]. This concept is particularly relevant in the field of geriatrics, but it also has applications in other vulnerable populations.

Social frailty—characterized by social isolation, limited social networks, and lack of social support—has been identified as an independent risk factor for adverse health outcomes, including disability and mortality [6, 7]. The social vulnerability index further demonstrates how social determinants interact with biological aging to shape health trajectories in older populations [8, 9].

Critically, frailty is not a static state but follows dynamic trajectories over time. Community-dwelling older adults transition between frailty states, with movements toward both worsening and improvement documented longitudinally [10]. These trajectories are shaped by multiple interacting factors, including medical conditions, functional status, social support, and environmental context [11]. Understanding these transitions is essential for identifying modifiable risk factors and intervention opportunities. However, while social conditions have been shown to influence frailty trajectories, the specific mechanisms through which changes in social and economic circumstances affect health outcomes—particularly hospitalization—remain insufficiently explored. Although there is strong evidence linking social isolation to negative health outcomes [12, 13], the effectiveness of interventions to reduce social isolation remains uncertain and understudied.

Hospitalization represents one of the most significant areas of expenditure within health systems. Individuals aged over 75 constitute the primary users of hospital services; this demographic is also the fastest-growing segment of the population, having increased by 12% in 2019. In Italy, this age group exhibits the highest hospitalization rate—228.79 per 1000 inhabitants in 2023—which is more than double that of the 65–74 age group. Moreover, their average length of hospital stay ranges from 9.3 to 9.7 days per admission, ∼20% longer than that of patients aged 65–74 [14].

These conditions contribute to substantially higher and increasing healthcare expenditures—∼40% more than those incurred by individuals aged 65–74, totaling an estimated 8 billion euros in 2022 [14]. Moreover, they often lead to patterns of hospital utilization that are not always conducive to the maintenance of optimal health. This is particularly concerning in light of the potential adverse effects associated with hospitalization itself, such as nosocomial infections and pressure ulcers, which pose significant health risks for all inpatients, and especially for older adults [15, 16].

Hospitalization is associated with increased psycho-physical and social frailty [17], as well as a higher incidence of the adverse outcomes linked to frailty itself [17]. Frailty has been consistently identified as a predictor of hospitalization in community-dwelling older adults [18], and frailty status at hospital admission predicts multiple adverse outcomes, including prolonged length of stay and mortality [19]. However, there is currently no conclusive evidence indicating whether reducing frailty—regardless of its specific nature—in community-dwelling individuals can effectively reduce hospitalization rates [20]. Moreover, frailty management is often narrowly interpreted as addressing only psycho-physical dimensions, while socio-economic frailty is frequently overlooked [20]. Research in this area remains limited, despite ongoing efforts to implement structured protocols for frailty management within hospital settings—protocols whose effectiveness has yet to be fully assessed [21, 22].

This study addresses these gaps by examining associations between trajectories of biopsychosocial frailty—with particular attention to changes in social and economic dimensions—and hospitalization rates among community-dwelling adults aged 75 and over. Specifically, we investigate whether improvements or deteriorations in living arrangements, informal support networks, social relationships, and financial conditions are independently associated with hospitalization risk. By analyzing longitudinal changes in these multidimensional frailty components, this study extends previous research that has primarily examined static frailty states or focused predominantly on physical frailty dimensions.

## Methods

This work stems from a secondary analysis of data already collected over the years to discuss the relationship between different components of frailty, social interventions, and hospitalization. It is a retrospective cohort study to analyze the impact of the trajectories of different components of biopsychosocial frailty on hospital acute admissions.

The data were obtained through the “Long Live the Elderly!” program, a community-based proactive monitoring initiative designed to counteract social isolation among individuals aged 80 and over living in the community. Individuals with an age between 75 and 80 could be included under request in the program. This program, previously described in other publications [23], is implemented by the Community of Sant’Egidio, a non-profit organization, under the framework of a memorandum of understanding with the municipal administrations of various cities. Based on the assessment of biopsychosocial frailty, the program provides a pro-active phone monitoring and a wide range of services divided into four groups:

1. Interventions (heat emergency, cold emergency, COVID-19 emergency, various interventions);
2. Primary prevention (vaccination information campaign and immunization support);
3. Monitoring (phone calls and/or home visits); and
4. Social networking (events, feasts, birthday wishes).

The data analysis is based on serial assessments of frailty conducted between 2016 and 2024, utilizing the Short Functional Geriatric Evaluation (SFGE) questionnaire [24] (Supplementary material SN.1), which is made up by 12 questions. This instrument categorizes individuals into four risk classes based on the total score: *Robust* (score = 0), *Pre-Frail* (score 1–2), *Frail* (score 3–9), and *Very Frail* (score >9), with increasing scores indicating a higher risk of adverse outcomes. The study population includes all individuals who underwent at least two frailty assessments and for whom at least one record of service provision was available. For each individual, only the first and most recent assessments were considered—regardless of the total number of evaluations—to determine changes in frailty status over time, classifying individuals as *improved*, *worsened*, or *stable* relative to their initial frailty class. This decision was made consistently with the objective of the analysis presented in this study: the aim was not to document the entire trajectory of individual frailty, but rather to collect evidence on the potential impact of changes in biopsychosocial frailty—and in specific components of this dimension—on the use of hospital services. The findings of this study provide the basis for further development of the analysis, which will consist of assessing the relevance of individual state transitions over an observation period averaging > 4 years.

The same procedure has been used to categorize changes in each item of the questionnaire according to the score variation. Psycho-physical status has been assessed as a unique variable cumulating the score of variable N.6 plus variables from N.8 to N.12. These variables have already been gathered as per the analysis of latent components of the questionnaire [25, 26]. The questions N.3, N.4, N.5, and N.7, concerning the socio-economic status and the social relationships, have been analyzed separately for their impact on hospitalization. Question N.1 (age) has been considered a control variable in the multivariable model and Question N.2 (education) which did not change substantially during the observation, has been excluded because its baseline value already contributed to the total frailty score.

Mean hospitalization rates were compared across frailty levels and socio-economic single items of the questionnaire. To assess the statistical significance of observed differences, both parametric and nonparametric tests, along with negative binomial regression models, were employed.

The anonymized data used for the secondary analysis conducted in this study are the result of interviews conducted by LLE program staff following the obtainment of informed consent. The use of the collected variables was evaluated and approved by the ethical committee (Ethical Committee of the University of Rome Tor Vergata R.S. 60/17).

## Results

The study sample comprised 6804 participants, of whom 718 were excluded from the analysis due to missing data. The final analytic sample included 6086 individuals, 65.9% of whom were women. Participants were followed for an average duration of 4.8 years (SD ± 1.5), with a total of 28 133 person-years of observation. The mean age of participants at baseline was 83.6 years (SD ± 4.9), with a median age of 83.1 years (interquartile range [IQR] 80.3–86.8) (Table 1). Each participant underwent an average of 3.3 frailty assessments (median = 3; IQR 2–4).

**Table 1.** Descriptive statistics

Characteristic	Overall *N* = 6086^a^	Robust *N* = 2463^a^	Pre-frail *N* = 1575^a^	Frail *N* = 1646^a^	Very frail *N* = 402^a^
Age (years)	83.0 (80.3, 86.8)	81.5 (79.4, 84.2)	83.8 (80.9, 87.4)	84.8 (81.0, 88.1)	86.4 (82.7, 90.3)
Origin	–	–	–	–	–
BRINDISI	187 (3.1%)	41 (1.7%)	49 (3.1%)	71 (4.3%)	26 (6.5%)
GENOVA	480 (7.9%)	172 (7.0%)	125 (7.9%)	157 (9.5%)	26 (6.5%)
NAPOLI	1480 (24.3%)	493 (20.0%)	361 (22.9%)	512 (31.1%)	114 (28.4%)
NOVARA	893 (14.7%)	384 (15.6%)	293 (18.6%)	185 (11.2%)	31 (7.7%)
ROMA	2886 (47.4%)	1312 (53.3%)	697 (44.3%)	685 (41.6%)	192 (47.8%)
SASSARI	160 (2.6%)	61 (2.4%)	50 (3.2%)	36 (%2.2)	13 (3.2%)

a Median (Q1, Q3); *n* (%).

Approximately 26% of participants were hospitalized at least once during the observation period, with about half of these experiencing multiple admissions (Table 2). The overall hospitalization rate was 125 per 1000 person-years (SD +/- 311, 95% CI:117-132).

**Table 2.** Acute hospital admissions

Admissions	No. of participants	%	Cumulative %	Total no. of admissions
0	4518	74.2	74.6	0
1	848	13.9	88.1	848
2	340	5.6	93.7	680
3	180	2.9	96.7	540
4	91	1.5	98.3	364
≥5	109	1.7	100.0	692
Total	6086	100.0	–	3124

Hospital admission rates increased progressively according to the level of biopsychosocial frailty, ranging from 94‰ (95% CI: 85–104) among Robust individuals to 110‰ (95% CI:98–123) among those who were classified as pre-frail, 175‰ (95% CI: 156–195) among the frail, and 157‰ (95% CI: 120–194) among the very frail (*P* < 0.001). Recurrent hospitalizations were likewise significantly associated with the degree of biopsychosocial frailty across all assessment points during the observation period (*P* < 0.001).

**Figure 1.**
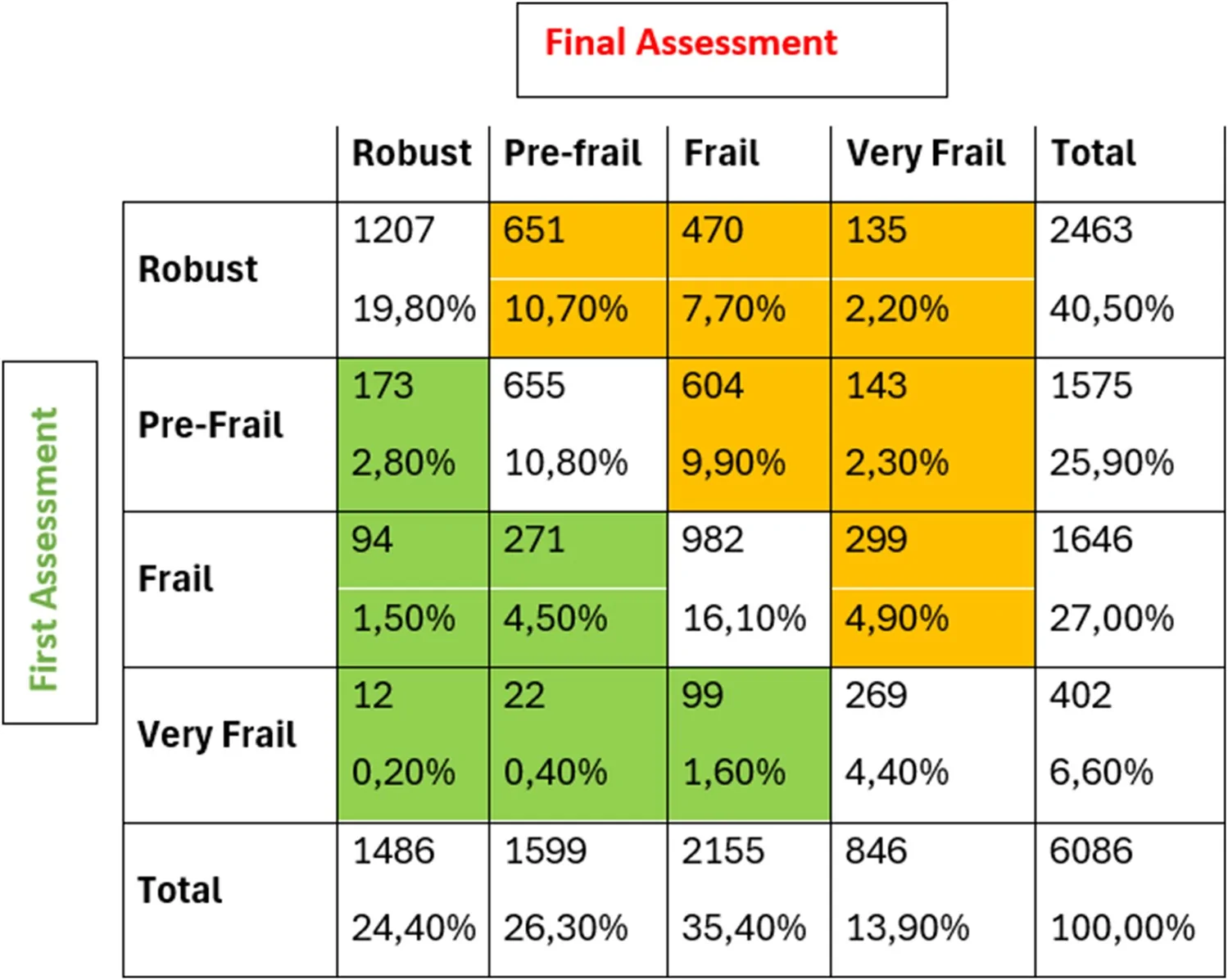
Distribution of biopsychosocial frailty according to the timing of assessment. White cells: enrolled subjects who showed the same level of frailty at both the first and last assessments. Yellow cells: enrolled subjects who showed a worsening of frailty at the last assessment compared to the first. Green cells: enrolled subjects who showed an improvement in frailty at the last assessment compared to the first.

The trajectories of each socio-economic item of the SFGE are summarized in Fig. 1 and Table 3. In Fig. 1, the yellow cells show the number and percentage of people who experienced a worsening of their biopsychosocial frailty over time, while the green cells show those who experienced an improvement. The percentage of individuals who maintain the same frailty class from the beginning to the end of the observation period (white cells) ranges approximately between 40% and 60% across all classes. In this case, it is difficult to determine the value of this element, as this result may partly be due to the action of the program, which tends to counteract state changes, especially in a worsening direction.

**Table 3.** Trajectories of SFGE socio-economic items

Q.N.3—Living arrangement		
–	*N*	%
Stable with partner	2186	35.9
Stable with others	965	15.9
Improved^a^	658	10.8
Moderately worsened^b^	247	4.1
Remained alone	573	9.4
Persistently alone	1447	23.8
Missing	10	0.2
Total	6086	100.0

Q.N.4—Informal network		
–	*N*	%
Permanently supported	3861	63.4
Support compromised	475	7.8
Support restored	905	14.9
Permanently unsupported	835	13.7
Missing	10	0.2
–	6.086	100.0

Q.N.5—Social relationship		
–	*N*	%
Improved	258	4.2
Stable	5340	87.7
Worsened	480	7.9
Missing	8	0.1
–	6086	100.0

Q.N.7—Financial situation		
–	*N*	%
Improved	584	9.6
Stable	5086	83.6
Worsened	407	6.7
Missing	9	0.1
–	6086	100.0

a Living arrangement “improved”: individuals who shifted from being alone to living with others.

b Living arrangement “Moderately worsened”: individual who shifted from partner to others.

The most notable improvement during the observation was recorded in the Q.N.4 (informal network—support restored, 14.9%) and Q.N.3 (living arrangement improved 10.8%). Over half of the individuals remained stable—defined as maintaining the same assessment level both at baseline and follow-up questionnaires—in all items. The highest proportion of worsening occurred in the Q.N.3 (living arrangements—“moderately worsened” plus “remained alone,” 13.5%) and Q.N.4 (informal network support compromised, 7.8%).

Univariable and multivariable analyses, including age and initial frailty status as control variables, showed an independent association between all socio-economic variables. A variation in hospital admissions, which achieved statistical significance in most of the cases, has been observed through the different trajectories within the questions’ answers (Table 4).

**Table 4.** Univariable and multivariable analysis (negative binomial regression) of the determinants of hospitalization

–	Univariable					Multivariable				
–	–	–	CL 95%		–	–	–	CL95%		
–	*P*	Rate ratio	Lower	Upper	Chi-square	*P*	Rate ratio	Lower	Upper	Chi-square
Q.N.3—Living arrangement	–	–	–	–	–	–	–			
Permanently with the partner	−0.067	0.936	0.853	1.026	0.001	0.439	1.551	1.278	1.881	<0.001
Permanently with others	0.378	1.460	1.304	1.635		0.442	1.555	1.233	1.962	
Improved	−0.073	0.930	0.821	1.052		−0.553	0.575	0.444	0.745	
Moderately worsened	−0.278	0.757	0.625	0.917		−0.366	0.694	0.480	1.003	
Remained alone	−0.001	0.999	0.877	1.137		0.112	1.118	0.875	1.430	
Persistently alone	Reference	1	–	–	–	Reference	1			
Q.N.4—Informal support	–	–	–	–	–	–	–			
Permanently supported	−0.132	0.876	0.795	0.965	< 0.001	−0.762	0.467	0.374	0.583	<0.001
Support compromised	−0.301	0.740	0.643	0.852		−0.792	0.453	0.339	0.605	
Support restored	0.052	1.054	0.936	1.186		−0.636	0.529	0.409	0.685	
Permanently unsupported	Reference	1	–	–	–	Reference	1			
Q.N.5—Social relationship	–	–	–	–	–	–	–			
Improved	0.510	1.665	1.366	2.031	< 0.001	0.588	1.800	1.207	2.686	<0.001
Stable	0.609	1.838	1.629	2.074		0.390	1.478	1.183	1.845	
Worsened	Reference	1	–	–	–	Reference	1			
Q.N.7—Financial situation	–	–	–	–	–	–	–			
Improved	−0.204	0.815	0.702	0.947	< 0.001	−1.428	0.240	0.175	0.330	<0.001
Stable	−0.364	0.695	0.619	0.780		−0.878	0.415	0.323	0.534	
Worsened	Reference	1	–	–	–	Reference	1			
–	–	–	–	–	–	–	–			
SFGE Frailty score	0.077	1.080	1.071	1.089	< 0.001	0.272	1.313	1.287	1.339	<0.001
Age (years)	0.022	1.022	1.014	1.030	< 0.001	0.050	1.051	1.035	1.068	<0.001
Psycho-physical status changes	0.307	1.359	1.315	1.405	< 0.001	0.675	1.964	1.844	2.091	<0.001

Outcome variable: No of Hospitalization; offset variables: semester of observation; continuous control variables: baseline SFGE frailty score and age, and changes in psycho-physical status during observation. I remain available for any further information.

Q.N.4 and Q.N.7 show a unidirectional and statistically significant relationship between improving trajectories and a lower number of hospitalizations: specifically, an improved or stable financial condition compared to the initial assessment, is associated with a reduction in the risk of hospitalization ranging from 58% to 76% (rate ratio [RR] = 0.240–0.415) compared to individuals whose financial situation worsened over the observation period.

Similarly, stable, restored, or even compromised social support compared to the assessment conducted at the beginning of the observation period is associated with a reduction of the hospitalization risk ranging from 37% to 21% (RR = 0.636–0.792) with respect to the individuals permanently unsupported.

The relationship between type of cohabitation (Q.N.3) and hospitalization risk appears more complex: the risk of hospitalization decreases for those who gave up living alone during the observation period (“improved”), while it increases for those who remained in the same condition during the entire observation period, compared to the risk of those who consistently lived alone.

Finally, the risk appears to be clearly increased for those who increased or maintained their social relationships during the observation period, compared to those whose social relationships decreased (Q.N.5).

As expected, worsening psycho-physical health status is associated with an increased risk of hospitalization. The same result is shown in case of an increase in frailty score and aging.

## Discussion

The article provides a valuable insight into the relationship between biopsychosocial frailty and hospital admissions among older adults, addressing multiple dimensions of frailty—socio-economic status, physical health, and social relationships—and their varying associations with hospitalization rates [27].

The study reveals that changes in the level of frailty components are associated with changes in admission rates. Notably, participants who showed stable or improved physical status, social support, or economic conditions had a lower risk of hospital admission than those who worsened. This finding suggests that maintaining or improving socio-economic conditions may be associated with reduced hospitalization risk, independent of physical frailty status.

It is evident that any change in frailty components poses a challenge to individuals, potentially leading to either deterioration or improvement, partly depending on environmental responses. Notably, a significant reduction in the hospitalization risk was observed among individuals who have been “permanently supported” by the social environment—a key focus of the “Long Live the Elderly!” program, in which all study participants were enrolled from. With regard to financial conditions, the main intervention implemented by the program facilitated the obtainment of financial benefits provided by the state to those with severe physical impairment or advanced cognitive decline. Although it is highly plausible that a portion of the observed reduction in hospitalizations is attributable to the program’s efforts to mitigate social isolation—the core aim of “Long Live the Elderly!”—or financial difficulties, the data do not allow for a distinction between the program’s effects and those stemming from informal support networks, such as family-initiated assistance. For example, where participants experienced increased physical decline requiring support with daily activities, family members may have independently provided additional care or financial support, irrespective of the program.

The association between better (improved) social and/or economic status and a subsequent reduction in hospitalization risk remains robust, suggesting a potential decrease of at least 20% annually among individuals over the age of 75. Especially, the robust and pre-frail individuals, characterized by minimal psycho-physical impairment, are likely to benefit the most from preventive interventions. Importantly, robust and pre-frail individuals account for roughly 50% of all hospital admissions in the sample, indicating that efforts to maintain or reduce frailty levels in this group could substantially lower hospitalization rates.

On the other hand, among the elderly, social isolation is considered a risk factor for both hospitalization and emergency room use, regardless of the psycho-physical situation. A large study conducted in the United States shows a 17% increase in hospitalization risk in those who have experienced conditions of social isolation in the previous 12 months even on a few occasions, even though without any change of psycho-physical situation and demographic parameters [28]. If we consider only Question 4 of the questionnaire regarding having someone to count on, the hospitalization rate increases from 97 hospitalizations per 1000 person-years in those who are permanently supported to 143 (an increase of ∼50%) in those declaring they are permanently unsupported (data not shown). These data represent a general indication of the impact that the lack of social support can have on the health of patients and on the use of hospital services, which are currently under strong pressure because of the progressive aging of the population along with the growth of multimorbidity and the related demand for health care.

The surprising observation that an increase in social relationships was associated with an increase in hospital admissions raises questions about the underlying factors driving this trend. It is important to distinguish between social support and social relationships. The former refers to tangible assistance in daily life, both psychological and practical, while the latter may consist of more or less superficial acquaintances who may or may not provide effective support. Social relationships help prevent isolation, and the well-known effect of increased survival associated with them may also be mediated by greater use of health services (including hospital admissions) thanks to the “intervention” of other people, even though they are not available for more structured support. However, social relationships are often less oriented toward providing concrete assistance and may simply represent social contacts that are sufficiently relevant to advise or suggest considering the worsening of a disease. Further research is needed to untangle these dynamics and identify the specific mechanisms through which social support, or its absence, affects health outcomes [29].

One of the main limitations of this study is that intermediate frailty states are not taken into account, nor the time spent in each frailty state or the events that occurred during the observation period that could have played a role in the evolution of frailty trajectories. This information should be the starting point for more in-depth analyses to be carried out with different statistical tools such as, for example, Markov models. Other limitations are related to the lack of clinical information that could highlight the reasons for the occurrence of more frequent hospital admissions. This lack is intrinsic to the questionnaire, even if the instrument maintains an effective representation of the individual risk of negative events, including hospitalization [24].

Additional limitations include the absence of adjustment for potentially confounding factors such as multimorbidity burden, baseline functional limitations, cognitive status, and level of engagement with the program itself. Furthermore, all participants were enrolled in a community-based monitoring program; findings may not generalize to older adults without access to such programs or to those who declined participation. Selection into and retention in the program may reflect unmeasured characteristics associated with both frailty trajectories and healthcare utilization.

The study suggests the need for a more integrated approach to managing frailty. While physical and socio-economic interventions have shown promise, the social dimension remains underexplored and often neglected in care strategies. The World Health Organization estimated ∼870 000 annual deaths due to social isolation worldwide [13]. Strengthening social networks and fostering meaningful connections could play a crucial role in enhancing overall well-being and reducing hospital dependency.

The study findings point out the potential of community-based interventions in reducing hospital admissions among older adults by addressing biopsychosocial frailty. In fact, in the framework of a community-based proactive monitoring program focused on counteracting social isolation like the “Long Live the Elderly!” program, reducing social isolation and improving financial conditions led to a reduction of hospital admission risk. To optimize these efforts, a more holistic approach including targeted social support alongside physical and economic assistance is essential. Future studies should focus on refining intervention strategies and evaluating their long-term impact on health outcomes, with particular attention to the nuanced relationship between social isolation and healthcare utilization [30].

## Supplementary Material

ckag011_Supplementary_Data

## Data Availability

Data supporting the findings of this study are available from the corresponding author upon request. Key points This study is among the first to quantify how changes in biopsychosocial frailty, particularly social and economic dimensions, influence hospitalization risk in community-dwelling older adults. It provides evidence that improved or stable financial conditions and informal social support significantly reduce hospital admissions, independent of physical frailty. The findings highlight that robust and pre-frail individuals, often overlooked in preventive care, account for nearly 50% of all hospitalizations, underscoring the value of early intervention. The study supports the role of community-based monitoring programs in reducing healthcare utilization by mitigating social isolation and supporting economic stability. It calls for a holistic public health strategy integrating social, economic, and physical dimensions of frailty to reduce avoidable hospitalizations in aging populations.
