# Supplementary material for: Social support and hospitalization in the elderly: investigating the role of frailty trajectories
Source: Eur J Public Health. 2026 Feb 16;36(2):ckag011. doi: 10.1093/eurpub/ckag011 (PMC13017486; doi:10.1093/eurpub/ckag011)
Supplement: ckag011_Supplementary_Data [file ckag011_supplementary_data.docx]

**Supplement 1:** Short Functional Geriatric Evaluation questionnaire (SFGE).

**ID ………………. Personal data:** Name………………………………………………………………………………………………

Gender…………. DoB …………………. Weight ………. Height ……….

1. Age

<75 (0)

75-85 (1)

>85 (2)

1. Education

None/Primary (1)

Secondary /Degree (0)

**Cohabitants**

1. Living ……..

Alone (1)

With Spouse (-1)

- - 1. Age of spouse: <75 (-1) 75-85 (0) >85(1)

With a paid assistant (0)

Others (0)

**Informal/formal social network**

1. In case of need, is there someone you can count on?
   1. Yes, for as long as necessary (-1)
   2. Yes, occasionally (0)

*(He/she would take me to the doctor 1 time or bring me lunch)*

- 1. No (1)

1. Are you involved in social activities or groups? Yes (-1) No (0)
2. Are you receiving formal care services? Yes (-1) No (0)

*(like Home care or participating in a daily center or meals on wheels or others)*

**Economic situation**

1. Your pension is enough to get to the end of the month?
   1. Yes (-1)
   2. With difficulty (0)
   3. No (1)
2. If it is not enough, why have you had problems in the last month?
   1. To buy food or medicine or clothes, to pay bills, or to pay

a person to help you in the Activities of Daily Living (2)

- 1. To pay for domestic help (1)

**Psychological condition**

1. Energy and Motivation Normal (0) Hypoactive/Hyperactive (2)

**Health/functional status**

1. Able to use the shower or bath independently Yes (0) No (2)
2. Gets out from the house Yes (0) No (2)
3. Bedridden Yes (4) No (0)
4. Confused Yes (8) No (0)
